# Super-Resolution Deep Learning Reconstruction for T2*-Weighted Images: Improvement in Microbleed Lesion Detection and Image Quality

**DOI:** 10.1007/s10278-025-01522-6

**Published:** 2025-04-29

**Authors:** Yusuke Asari, Koichiro Yasaka, Kazuki Endo, Jun Kanzawa, Naomasa Okimoto, Yusuke Watanabe, Yuichi Suzuki, Shiori Amemiya, Shigeru Kiryu, Osamu Abe

**Affiliations:** 1 Department of Radiology, Graduate School of Medicine, The University of Tokyo, 7 - 3- 1 Hongo, Bunkyo-Ku, Tokyo, 113 - 8655 Japan; 2 Department of Radiology, International University of Health and Welfare Narita Hospital, 852 Hatakeda, Narita, Chiba 286 - 0124 Japan

**Keywords:** Microbleed, Image quality, Magnetic resonance imaging

## Abstract

Super-resolution deep learning reconstruction (SR-DLR) is a promising tool for improving image quality by enhancing spatial resolution compared to conventional deep learning reconstruction (DLR). This study aimed to evaluate whether SR-DLR improves microbleed detection and visualization in brain magnetic resonance imaging (MRI) compared to DLR. This retrospective study included 69 patients (66.2 ± 13.8 years; 44 females) who underwent 3 T brain MRI with T2*-weighted 2D gradient echo and 3D flow-sensitive black blood imaging (reference standard) between June and August 2024. T2*-weighted images were reconstructed using SR-DLR and DLR. Three blinded readers detected microbleeds and assessed image quality, including microbleed and normal structure visibility, sharpness, noise, artifacts, and overall quality. Quantitative analysis involved measuring signal intensity along the septum pellucidum. Microbleed detection performance was analyzed using jackknife alternative free-response receiver operating characteristic analysis, while image quality was analyzed using the Wilcoxon signed-rank test and paired *t*-test. SR-DLR significantly outperformed DLR in microbleed detection (figure of merit: 0.690 *vs.* 0.645, *p* < 0.001). SR-DLR also demonstrated higher sensitivity for microbleed detection. Qualitative analysis showed better microbleed visualization for two readers (*p* < 0.001) and improved image sharpness for all readers (*p* ≤ 0.008). Quantitative analysis revealed enhanced sharpness, especially in full width at half maximum and edge rise slope (*p* < 0.001). SR-DLR improved image sharpness and quality, leading to better microbleed detection and visualization in brain MRI compared to DLR.

## Introduction

Microbleeds are associated with several etiologies including cerebral amyloid angiopathy, hypertensive encephalopathy, diffuse axonal injury, etc. In addition to these, other etiological factors have been gaining wide attention recently. One of the hypothesized etiological factors for Alzheimer’s disease is the abnormal accumulation of amyloid beta species within the brain [1, 2]. Recent advancements in therapeutic approaches have increasingly focused on this pathological mechanism [3]. Amyloid-related imaging abnormalities (ARIAs), which are adverse events associated with anti-amyloid beta therapies, have been reported in several Alzheimer’s disease trials [4]. ARIAs can be observed via MRI in two radiographically different presentations: ARIA-E and ARIA-H. ARIA-E refers to signal abnormalities believed to represent vasogenic edema, whereas ARIA-H refers to changes attributable to microbleeds and hemosiderosis [5–8]. Two general approaches—T2*-weighted gradient echo sequences (GRE) and susceptibility weighted imaging, are used to detect ARIA-H, with susceptibility weighted imaging believed to detect lesions more precisely [8]. However, susceptibility weighted imaging is limited to a few facilities, and there is variation among MRI device vendors [9].

Recent advances in deep learning technology have been remarkable, and various applications in radiology, including image reconstruction and diagnosis, have been reported [10, 11]. Methods involving deep learning reconstruction (DLR) have been shown to decrease noise and improve image quality in computed tomography and MR images [12–18]. Super-resolution DLR (SR-DLR) has also emerged, which can further enhance the spatial resolution of MR images in addition to conventional DLR [19–22]. Cervical spine neuroforamen and cranial nerves are examples of microstructures for which SR-DLR has demonstrated its utility [21, 22]. This new technology is expected to make ARIA-H, particularly microbleeds, more visible because they are sometimes difficult to detect even in high-quality images because of their small size. Previous studies on deep learning techniques for microbleeds have demonstrated a reduction in acquisition time and an enhancement in image quality for susceptibility-weighted imaging, but not for T2*-weighted GRE [23, 24]. To the best of our knowledge, no studies have addressed SR-DLR and microbleeds, or deep learning techniques and T2*-weighted GRE.

This study aimed to assess whether SR-DLR improves the detection performance and visualization of microbleeds in brain T2*-weighted images compared with conventional DLR.

## Materials and Methods

### Study Design

This retrospective single-center study was approved by the Institutional Review Board of our institution, which waived the need for written informed consent due to the retrospective nature of the study.

**Fig. 1:**
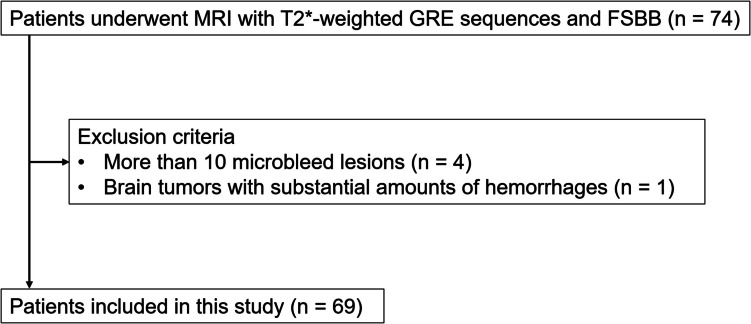
Patient inclusion flowchart. GRE, gradient echo. FSBB, flow sensitive black blood imaging

Consecutive patients who underwent brain 3 T MRI with axial T2*-weighted 2D GRE and 3D flow sensitive black blood imaging (FSBB), which is like susceptibility weighted imaging, sequences between June 2024 and August 2024 were considered for inclusion (Fig. 1). The exclusion criteria were as follows: individuals with > 10 microbleed lesions and those with brain tumors associated with substantial numbers of hemorrhages. The reasons for the exclusion criteria are that cases with a large number of microbleed lesions can become a physical burden for readers and influence the consistency of assessment.

### MRI Protocol

MRI was performed using a 3-T scanner (Vantage Centurian; Canon Medical Systems, Otawara, Japan). The axial T2*-weighted 2D GRE imaging parameters were as follows: repetition time, 690.0 ms; echo time, 10.0 ms; flip angle, 20°; echo train length, 1; acquisition matrix, 256 × 192; pixel bandwidth, 391 Hz; number of acquisitions, 1; and imaging time, 124 s. The images were reconstructed using SR-DLR (Precise IQ Engine; Canon Medical Systems) and DLR (Advanced Intelligent Clear-IQ Engine; Canon Medical Systems) with the following parameters (SR-DLR/DLR): slice thickness, 3 mm/3 mm; pixel spacing, 0.1736 mm/0.2734 mm; and row × column, 1152 × 1152/768 × 768. The axial 3D FSBB parameters were as follows: repetition time, 39.0 ms; echo time, 30.0 ms; flip angle, 18°; echo train length, 1; acquisition matrix, 432 × 304; pixel bandwidth, 89 Hz; number of acquisitions, 1; and imaging time, 194 s.

### Overview of DLR and SR-DLR Algorithm

In the DLR algorithm (Advanced Intelligent Clear-IQ Engine; Canon Medical Systems, commercially available), the data is initially processed using discrete cosine transform and separated into a zero-frequency component and high-frequency components. The high-frequency components are then processed through 12 convolutional layers for noise reduction and inverse discrete cosine transform. Images acquired with a high number of acquisitions are utilized as reference data in this process [25]. The SR-DLR algorithm (Precise IQ Engine; Canon Medical Systems, commercially available) operates in two stages: denoising and up-sampling. The denoised image, which results from the denoising step based on a neural network architecture similar to conventional DLR undergoes further processing via a fast Fourier transform. For the up-sampling stage of SR-DLR, zero-filling is applied to the k-space with a three-fold interpolation factor to improve spatial resolution. Afterward, an inverse fast Fourier transform is performed and a U-net-based neural network trained to reduce ringing artifacts from zero-filling is applied. During the training of SR-DLR for 3 T MRI, a dataset consisting of various body regions (e.g., brain, lumbar spine, knee), different contrasts (T1-weighted, T2-weighted, diffusion-weighted), and multiple planes (axial, coronal, sagittal) was used. The dataset contained 45936 MRI image pairs for denoising neural network and 46018 MRI image pairs for artifact-reduction neural network. Consequently, SR-DLR images are characterized by high resolution and minimal noise [19].

### Reference Standard

Microbleeds detectable by FSBB were considered true lesions. This determination was made based on a consensus between one board-certified radiologist with 15 years of imaging experience and one radiology resident with 3 years of imaging experience.

### Lesion Detection and Qualitative Image Analyses

A board-certified radiologist with 15 years of imaging experience randomized all image sets. Two additional radiologists (Readers 1 and 2, with 14 and 7 years of imaging experience, respectively) and one radiology resident (Reader 3, with 3 years of imaging experience) independently analyzed the T2*-weighted images using ImageJ (https://imagej.net/ij/). They were blinded to the patient details and reconstruction methods.

The readers documented all microbleed lesions (≤ 10 mm in diameter) and recorded the location. Furthermore, they recorded confidence scores on a four-point scale (4, definitely present; 3, probably present; 2, uncertain for the presence or absence; and 1, no lesion). The test was conducted in two sessions, and the SR-DLR and DLR images for the same patient were ensured not to appear in the same session. A 2-week interval was maintained between sessions to prevent recall bias.

The three same readers assessed the T2*-weighted images at a minimum of 2 weeks after the lesion detection test. The images were assessed using the following criteria:

- Microbleed lesion depiction (all lesions were annotated and assessed) (4, clear depiction; 3, slightly blurred; 2, moderately blurred; and 1, unrecognizable)
- Structure depiction (superficial cerebral veins and falx cerebri) (4, clear depiction; 3, slightly blurred; 2, moderately blurred; and 1, unrecognizable)
- Sharpness of images (4, sharp; 3, slightly blurred for a few structures; 2, moderately blurred; 1, blurring affecting imaging diagnosis)
- Subjective image noise (4, less noise; 3, standard noise; 2, more than standard noise; and 1, severe noise)
- Presence of artifacts (3, almost no artifacts; 2, mild artifacts; 1, severe artifacts affecting imaging diagnosis)
- Overall image quality (5, excellent; 4, better than standard; 3, standard; 2, worse than standard; and 1, poor)

Iron deposition in the basal ganglia can be used for qualitative analysis as a mimic of microbleed. However, since it may not be observed in young patients, it is difficult to evaluate it consistently. Thus, we decided to assess the superficial cerebral veins for structure depiction analysis, which are observable in all patients.

### Quantitative Image Quality Analyses

**Fig. 2:**
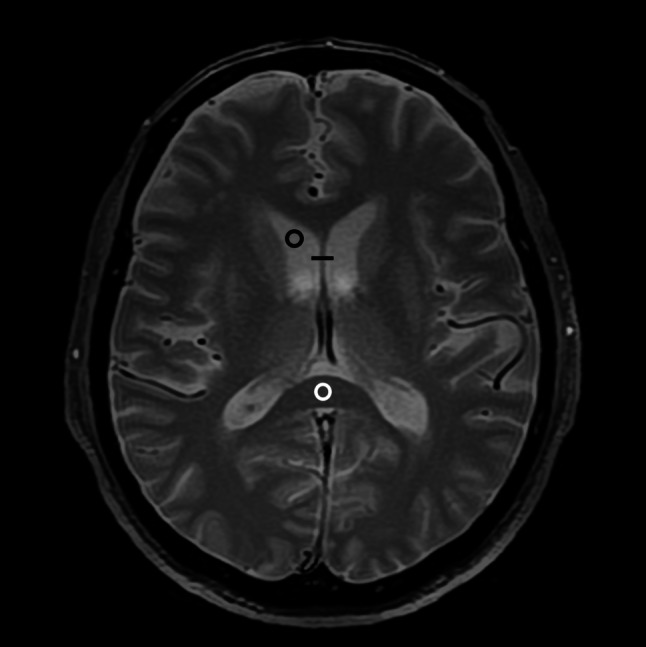
A linear region of interest (ROI) was located at the septum pellucidum (black line). Circular or ovoid ROIs were set on the splenium of the corpus callosum (white circle) and the right anterior horn of the lateral ventricle (black circle). ROI, region of interest

Quantitative image quality analyses were performed on the T2*-weighted images by a radiology resident with 3 years of imaging experience using ImageJ. A linear region of interest (ROI) passing through the septum pellucidum was placed (Fig. 2). The intensity profile of the signal along the linear ROI was subsequently recorded. The full width at half maximum (FWHM), edge rise distance (ERD), and edge rise slope (ERS) were measured and calculated. The ERD and ERS values were averaged across both edges. Lower values indicated better image sharpness for FWHM and ERD, whereas higher values indicated better sharpness for ERS [20, 26]. Furthermore, two circular or ovoid ROIs measuring 3–5 mm in diameter, located on the splenium of the corpus callosum and the right anterior horn of the lateral ventricle, were placed (Fig. 2). The mean and standard deviation of the signal intensity for each ROI were calculated and documented.

The following metrics were then computed:

$${\mathrm{SNR}}_{\mathrm{BRAIN}}={\mathrm{SI}}_{\mathrm{BRAIN}}/{\mathrm{SD}}_{\mathrm{BRAIN}}$$

$${\mathrm{SNR}}_{\mathrm{CSF}}={\mathrm{SI}}_{\mathrm{CSF}}/{\mathrm{SD}}_{\mathrm{CSF}}$$

$$\mathrm{CNR}=({\mathrm{SI}}_{\text{CSF }}-{\mathrm{SI}}_{\mathrm{BRAIN}})/\surd (({\mathrm{SD}}_{{\mathrm{BRAIN}}^{2}}+{\mathrm{SD}}_{{\mathrm{CSF}}^{2}})/2)$$

CNR, SD_BRAIN_, SD_CSF_, SI_BRAIN_, SI_CSF_, SNR_BRAIN_, and SNR_CSF_ refer to the contrast-to-noise ratio, standard deviation of the signal intensity for the brain and CSF, mean signal intensity for the brain and CSF, and signal-to-noise ratio for the brain and CSF, respectively.

The location and size of the ROIs were kept consistent between SR-DLR and DLR.

### Statistical Analysis

Statistical analyses were performed using R (version 4.3.1; https://www.r-project.org/). The *t*-test and Fisher’s exact test were used to compare demographic characteristics between the microbleed lesion and non-microbleed lesion groups. To assess the diagnostic performance for detecting microbleeds using the diagnostic confidence score, jackknife alternative free-response receiver operating characteristic analysis was performed using the R package “RJafroc.” This analysis provided the figure of merit (FOM), which is similar to the area under the curve in the traditional receiver operating characteristic analysis. To analyze the sensitivity of the detection test, diagnostic confidence scores of ≥ 2 were considered indicative of the presence of lesions. These scores were evaluated statistically using McNemar’s test. The qualitative and quantitative image analysis scores for SR-DLR and DLR were evaluated using the Wilcoxon signed-rank test and paired *t*-test, respectively. *P*-values < 0.050 were used to denote statistical significance.

## Results

### Patient Characteristics

The study population comprised 74 participants. Individuals who had > 10 microbleed lesions (4) and those with metastatic brain tumors associated with substantial amounts of hemorrhage (1) were excluded. Ultimately, 69 patients (average age, 66.2 ± 13.8 years; 25 males and 44 females) with 46 microbleed lesions (2 patients with 4 lesions, 4 patients with 3 lesions, 6 patients with 2 lesions, 14 patients with 1 lesion, and 43 participants with no lesion) were included in the study (Fig. 1). The sizes of the microbleed lesions were as follows: < 2.5 mm (30 lesions), 2.5–5.0 mm (14 lesions), 5.0–7.5 mm (1 lesion), and ≥ 7.5 mm (1 lesion). The locations were as follows: right frontal lobe (7 lesions), left frontal lobe (6 lesions), right parietal lobe (3 lesions), left parietal lobe (6 lesions), right temporal lobe (5 lesions), left temporal lobe (1 lesion), right basal ganglia (3 lesions), left basal ganglia (1 lesion), left insula (1 lesion), right thalamus (1 lesion), left thalamus (4 lesions), pons (1 lesion), right cerebellar (4 lesions), and left cerebellar (3 lesions). The microbleed lesion group was older (*p* = 0.026) and had a higher proportion of males (*p* = 0.005) than the non-microbleed lesion group (Table 1). Indications for MRI included cognitive decline (3), headache (3), syncope (2), hearing loss (1), gait unsteadiness (1), evaluation and follow-up of cerebrovascular disease (n = 41), evaluation and follow-up of intracranial tumors (10), follow-up of neck tumor (1), follow-up of demyelinating diseases (4), evaluation of meningitis (1), and follow-up after microvascular decompression surgery (2).

**Table 1:** Demographic characteristics of the patients in the microbleed lesion and non-microbleed lesion groups

	Microbleed lesion group (*n* = 26)	Non-lesion group (*n* = 43)	*P* value
Age (years: mean ± standard deviation)	70.9 ± 12.3	63.3 ± 14.0	0.026 *
Sex (male, female)	15, 11	10, 33	0.005 *

Age; *t*-test. Sex; Fisher’s exact test

### Lesion Detection

**Fig. 3:**
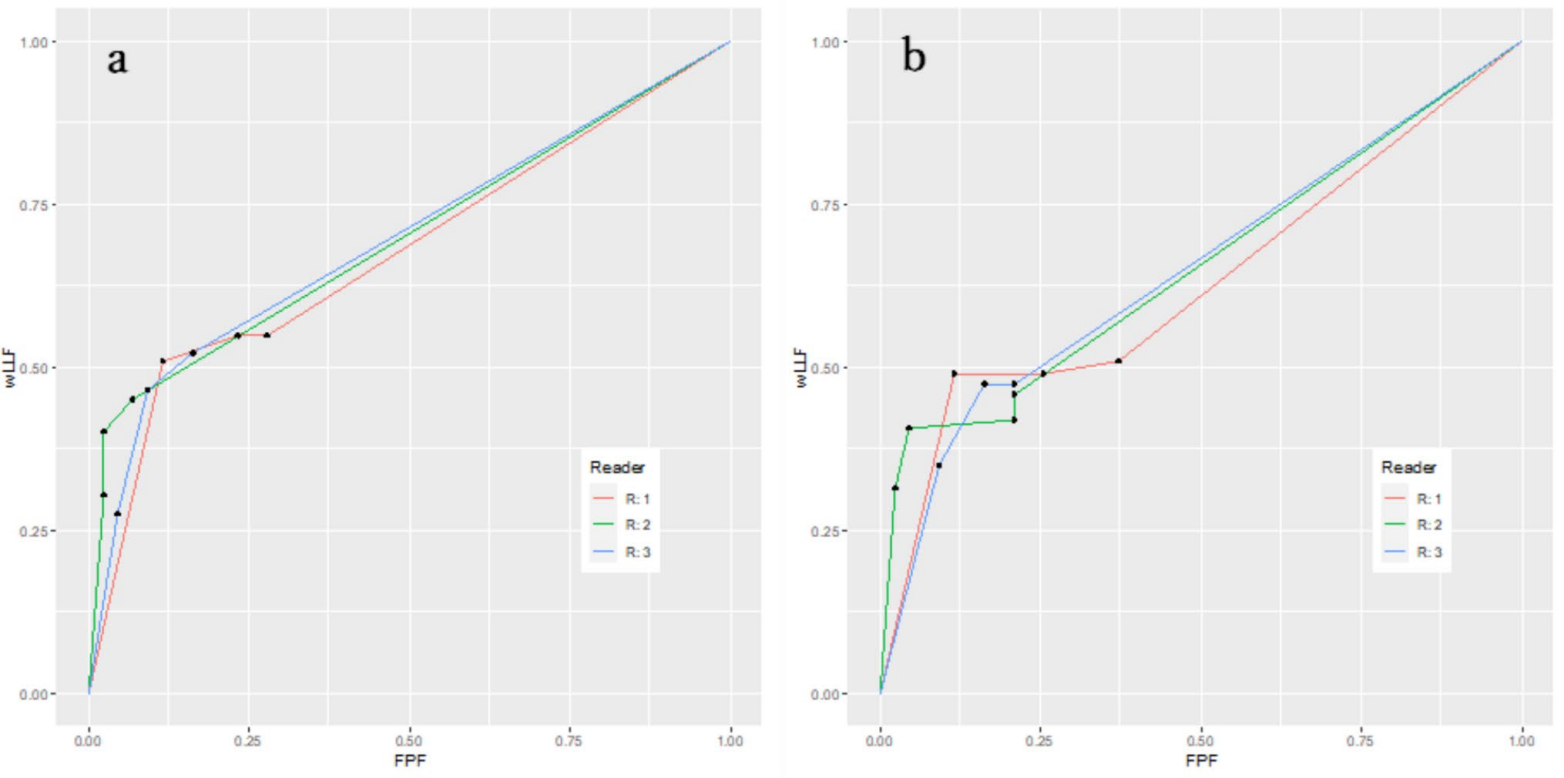
Free-response receiver operating characteristic curves for the detection of microbleeds in super-resolution deep learning reconstruction (**a**) and deep learning reconstruction (**b**). wLLF, weighted lesion localization fraction. FPF, false-positive fraction

Table 2 and Fig. 3 present the lesion detection results. The FOM for detecting microbleeds among the three readers was 0.690 in SR-DLR (with reader scores of 0.675, 0.695, and 0.699, respectively) and 0.645 in DLR (with scores of 0.629, 0.650, and 0.656, respectively). The three readers exhibited better performance in SR-DLR than in DLR for detecting microbleeds (*p* < 0.001).

**Table 2:** Lesion detection results

	Reader	SR-DLR	DLR	*P* value
Figures of merit	1	0.675	0.629	
	2	0.695	0.650	
	3	0.699	0.656	
	Mean (95% CI)	0.690(0.605–0.774)	0.645 (0.549–0.741)	< 0.001 *
Sensitivity	1	52%	48%	0.724
	2	46%	41%	> 0.99
	3	43%	41%	0.683
False-positive counts	1	39	45	
	2	18	23	
	3	9	20	

Figures of merit are based on the reader’s diagnostic confidence score with jackknife alternative free-response operating characteristic analysis. Sensitivity was compared with McNemar’s test

^*^Statistically significant difference

The microbleed detection sensitivity was 52%, 46%, and 43% for readers 1, 2, and 3, respectively, using SR-DLR. Although no statistically significant difference was observed (reader 1, *p* = 0.724; reader 2, *p* > 0.99; and reader 3, *p* = 0.683), these values were higher than those in DLR (48%, 41%, and 41% for readers 1, 2, and 3, respectively). The numbers of false-positive results were 39, 18, and 9 for readers 1, 2, and 3, respectively, in SR-DLR and 45, 23, and 20 for readers 1, 2, and 3, respectively, in DLR. The false-positive counts were lower for SR-DLR than for DLR.

### Qualitative Image Quality Analyses

**Fig. 4:**
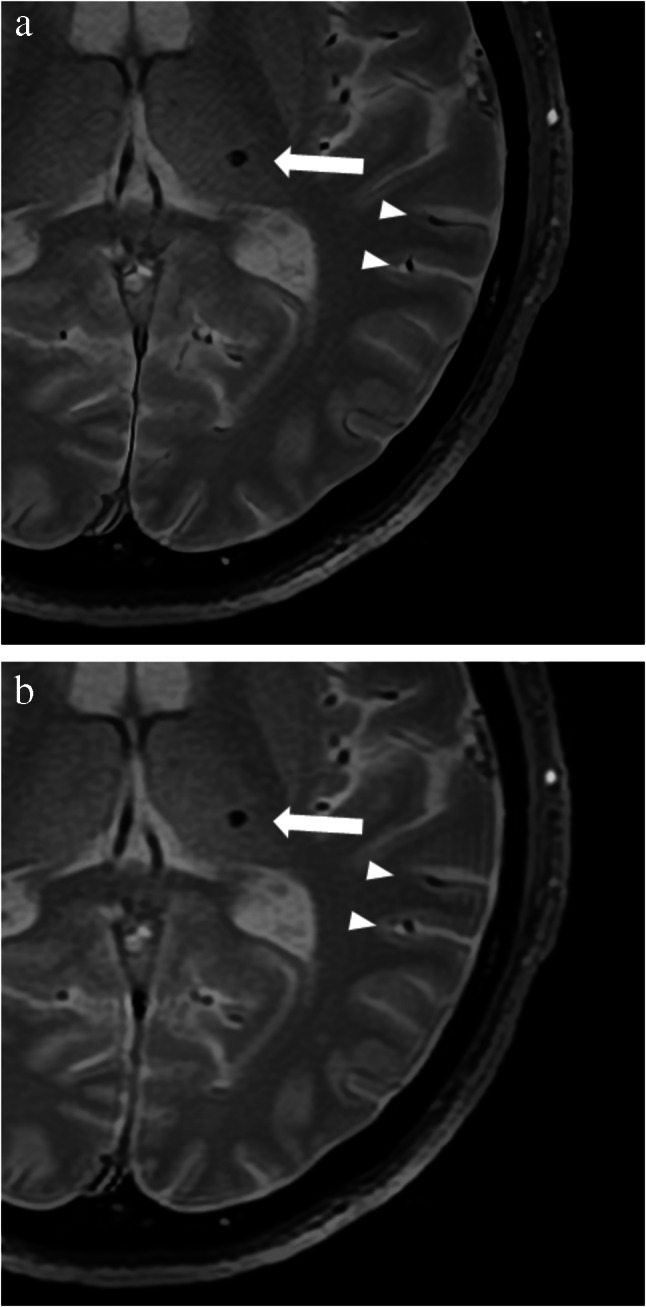
T2*-weighted MR images (super-resolution deep learning reconstruction (**a**) and deep learning reconstruction (**b**)) of a microbleed lesion (arrow) and superficial cerebral veins (arrowheads) of a 58-year-old male patient

**Fig. 5:**
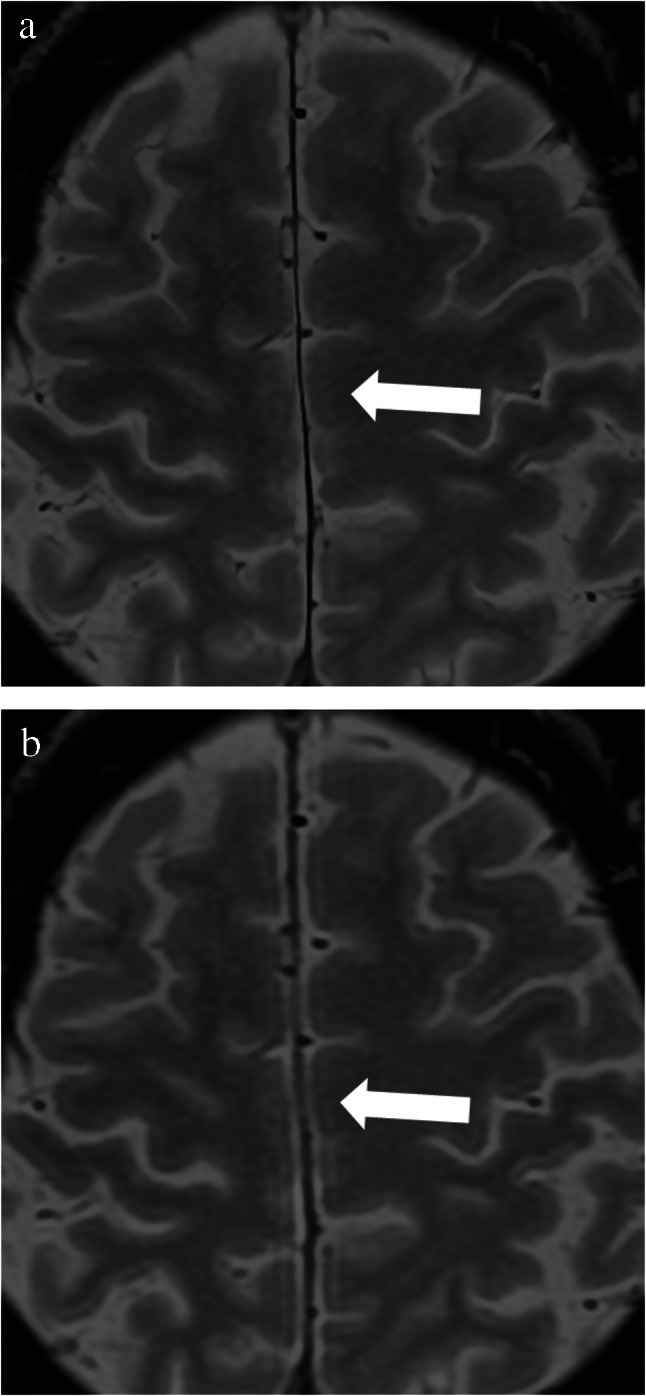
T2*-weighted MR images (super-resolution deep learning reconstruction (**a**) and deep learning reconstruction (**b**)) of the falx cerebri (arrow) of a 58-year-old male patient (same patient as Fig. 4)

Table 3 presents the results of the qualitative image quality analyses. The depiction of microbleed (Fig. 4) was rated better in SR-DLR than in DLR by readers 1 and 3 (*p* < 0.001). For the depiction of superficial cerebral veins (Fig. 4) and falx cerebri (Fig. 5), all readers rated significantly better in SR-DLR than in DLR (*p* ≤ 0.008), which was also true for the sharpness of images (*p* < 0.001). Readers 1 and 3 disagreed regarding the subjective image noise assessment because reader 1 rated DLR better (*p* < 0.001) and reader 3 rated SR-DLR better (*p* < 0.001). No significant difference was observed between DLR and SR-DLR according to reader 2 (*p* = 0.121). In the analysis of artifacts, readers 1 and 3 did not find a significant difference between SR-DLR and DLR (*p* = 0.074 and *p* = 0.574), whereas reader 2 found significantly more artifacts in SR-DLR (*p* = 0.035). The overall image quality was rated better in SR-DLR for all three readers (*p* ≤ 0.014).

**Table 3:** Qualitative image analysis results

	Reader	SR-DLR	DLR	Comparison
Microbleed lesion depiction	1	26/5/1/14	2/24/6/14	< 0.001 *
	2	17/13/2/13	20/7/7/11	0.953
	3	16/11/5/14	0/19/12/14	< 0.001 *
Structure depiction (superficial cerebral veins)	1	67/2/0/0	1/68/0/0	< 0.001 *
	2	49/18/2/0	31/35/3/0	0.008 *
	3	69/0/0/0	5/59/5/0	< 0.001 *
Structure depiction (falx cerebri)	1	68/1/0/0	0/69/0/0	< 0.001 *
	2	51/17/1/0	24/42/3/0	< 0.001 *
	3	69/0/0/0	6/60/3/0	< 0.001 *
Sharpness of images	1	68/1/0/0	0/69/0/0	< 0.001 *
	2	44/20/5/0	19/44/5/1	< 0.001 *
	3	69/0/0/0	2/63/4/0	< 0.001 *
Subjective image noise	1	8/61/0/0	62/7/0/0	< 0.001 *
	2	54/15/0/0	48/19/2/0	0.121
	3	66/3/0/0	23/43/3/0	< 0.001 *
Presence of artifacts	1	36/31/2	46/20/3	0.074
	2	39/30/0	51/17/1	0.035 *
	3	10/56/3	9/55/5	0.574
Overall image quality	1	66/3/0/0/0	0/65/4/0/0	< 0.001 *
	2	26/32/11/0/0	13/38/17/1/0	0.014 *
	3	49/19/0/1/0	0/38/29/2/0	< 0.001 *

The numbers of patients with each score (5/4/3/2/1, 4/3/2/1, or 3/2/1) are shown. DLR, deep learning reconstruction; SR-DLR, super-resolution deep learning reconstruction; *, statistically significant difference

### Quantitative Image Quality Analyses

Table 4 presents the results of the quantitative image quality analyses. For indicators related to image sharpness in SR-DLR, FWHM and ERS exhibited improvements over DLR (*p* < 0.001). ERD exhibited no significant difference. No significant difference in the SNR and CNR assessment was observed.

**Table 4:** Quantitative image analysis results

	SR-DLR	DLR	Comparison
FWHM (mm)	1.2 ± 0.5	1.8 ± 0.4	< 0.001 *
ERD (mm)	1.4 ± 0.4	1.5 ± 0.4	0.102
ERS (mm^−1^)	2277.2 ± 987.8	1707.2 ± 617.1	< 0.001 *
SNR_BRAIN_	28.1 ± 6.6	28.4 ± 6.3	0.579
SNR_CSF_	48.3 ± 11.7	47.8 ± 12.7	0.520
CNR	16.2 ± 3.4	16.1 ± 3.5	0.322

CNR, contrast-to-noise ratio, DLR, deep learning reconstruction, ERD, edge rise distance, ERS, edge rise slope, FWHM, full width at half maximum, SNR_CSF_, signal-to-noise ratio for CSF, SNR_BRAIN_, signal-to-noise ratio for the brain, SR-DLR, super-resolution deep learning reconstruction

^*^, statistically significant difference

## Discussion

This study aimed to evaluate whether SR-DLR can enhance the detection and visualization of microbleeds in brain MR images compared with conventional DLR. The background of the study is the challenge of detecting microbleeds using T2*-weighted GRE images. The key results of the lesion detection analysis indicated that SR-DLR significantly outperformed DLR in terms of the FOM (*p* < 0.001). In qualitative and quantitative image assessments, SR-DLR consistently yielded superior image quality across multiple criteria, including microbleed depiction, sharpness, and overall image quality for qualitative analysis and FWHM and ERS for quantitative analysis.

SR-DLR employs a deep learning model to initially reduce noise and enhance image quality through traditional DLR techniques. The output image is further processed via a fast Fourier transform. It then applies an enhanced spatial-resolution method involving zero-filling interpolation of the k-space, followed by an inverse fast Fourier transform and additional neural network processing to minimize artifacts from the zero-filling process. This two-step approach has been reported to improve spatial resolution [19, 21, 22].

In the qualitative analysis, readers 1 and 3 reported significantly better microbleed depiction performance in SR-DLR than in DLR. Previous studies have shown that SR-DLR can improve the visibility of small structures and lesions, such as cranial nerves and neuroforaminal stenosis [21, 22]. This is consistent with our results on microbleeds, which are also small lesions. All three readers, including reader 2, considered that SR-DLR could better depict other small structures, such as the cerebral veins and falx cerebri. The reason for reader 2 not finding a significant difference in microbleed depiction may be attributed to differences in individual experience, biases, or assessment habits, and reader 2 may have placed greater emphasis on other image quality factors.

The microbleed detection sensitivity tended to be higher with SR-DLR than with DLR. However, the detection sensitivity of SR-DLR was not very high. This may be because microbleeds that can be detected by FSBB were considered true lesions, which, in fact, includes several lesions that were too small to detect by T2*-weighted images but were only identifiable on FSBB, as is often the case with these two sequences [27]. Our study results will benefit patients undergoing brain MRI at institutions where FSBB is unavailable.

Image sharpness, which is also a strong point of SR-DLR, exhibited significant improvement the subjective evaluations, contributing to the enhancement of spatial resolution. Objectively, improvements in several spatial-resolution metrics, such as FWHM and ERS, were also observed with SR-DLR.

Generally, spatial resolution is negatively correlated with image noise. In this study, some variability was observed in the subjective assessment of image noise among the readers. The discrepancies could be due to differences in individual experience or subjective interpretations of image quality. However, objective noise metrics, such as SNR and CNR, exhibited no significant differences between SR-DLR and DLR, which are more precise indicators of noise detection. These findings suggest that although image noise may have a slight impact on image quality in SR-DLR, improved spatial resolution has a more substantial effect on overall image quality and lesion detection. Although no cases of ARIA were observed in this study, the improved detection and visualization of microbleeds may significantly enhance the diagnosis of ARIA-H. Furthermore, it can provide clinical benefits in other fields, such as stroke risk assessment, early diagnosis and monitoring of dementia, and monitoring of patients receiving antiplatelet or anticoagulant therapy.

Although the results of this study are promising, several limitations must be considered. First, the retrospective nature of the study may introduce patient selection bias. Second, the relatively small sample size may limit the generalizability of the findings. Future prospective studies with larger cohorts and more diverse patient populations are necessary to validate our findings and further explore the clinical utility of SR-DLR. Third, cognitive decline was an indication for MRI in only three cases in this study, with other indications, such as follow-up evaluations of aneurysms, accounting for a larger portion of the cases. Fourth, distinguishing microbleeds from cavernous hemangiomas is sometimes difficult on brain MRI, and no surgical operations were performed on the lesions. Thus, some lesions may contain cavernous hemangiomas. The time required for the reconstruction of T2*-weighted GRE images was longer for SR-DLR (approximately 27 s) compared to DLR (approximately 14 s) for each patient. However, it should be noted that a precise evaluation and comparison of reconstruction time were difficult due to technical limitations. As for the costs, it is important to note that commercial SR-DLR application may not be affordable at all facilities.

## Conclusions

This study demonstrates that SR-DLR significantly improves microbleed visualization on brain MRI compared with conventional DLR. Both qualitative and quantitative analyses revealed enhancements in image sharpness, lesion detection, and overall image quality with SR-DLR, particularly in the visualization of microbleeds and fine cerebral structures. Although sensitivity improvements were modest, the reduction in false-positive results and higher FOM suggest SR-DLR’s potential for improving diagnostic accuracy in detecting microbleeds such as in the context of monitoring ARIAs in Alzheimer’s disease. Further studies, including larger and more diverse patient cohorts, are required to validate these findings and refine SR-DLR algorithms for broader clinical applications.

## Data Availability

The datasets generated and/or analyzed during the current study are not publicly available due to patients' confidentiality.
